# Impact of COVID-19 vaccination and vaccine type on morbidity and mortality in hospitalized patients: a retrospective cohort study in Egypt

**DOI:** 10.3389/fmicb.2026.1792503

**Published:** 2026-02-27

**Authors:** Nehal Mohamed Eisa, Ramy M. El Sabaa, Shrouq Sayed Abdelrazik, Rana A. Hussein, Marwa M. Gaballah, Reem S. Mahmoud, Nourhan M. Kamal, Mostafa Ahmed, Ahmed Essam Abou Warda, Seif El Hadidi, Heba Khaled, Heba Salama, Haidy M. Sami, Mostafa M. Bahaa, Hayam Ali AlRasheed, Abdelrahman S. H. Refaee

**Affiliations:** 1 Clinical Research Department at Giza Health Affairs Directorate, MOHP, Giza, Egypt; 2 Biochemistry and Molecular Biology Department, Faculty of Pharmacy (Girls), Al-Azhar University, Cairo, Egypt; 3 Clinical Pharmacy Department, Faculty of Pharmacy, Menoufia University, Menoufia, Egypt; 4 Clinical Pharmacy Department, Faculty of Pharmacy, October 6 University, Giza, Egypt; 5 Clinical Pharmacy Department, School of Life and Medical Sciences, Hertfordshire, United Kingdom; 6 Department of Biochemistry, Faculty of Pharmacy, Cairo University, Cairo, Egypt; 7 Giza Memorial Institute for Ophthalmic Research, Giza, Egypt; 8 Department of Pharmacy Practice, Faculty of Pharmacy, Delta University for Science and Technology, Gamasa, Egypt; 9 Pharmacy Practice Department, Faculty of Pharmacy, Horus University, New Damietta, Egypt; 10 Pharmacy Practice Department, Faculty of Pharmacy, Mansoura National University, Gamasa, Egypt; 11 Pharmacy Practice Department, Faculty of Pharmacy, East Port Said National University, Port Said, Egypt; 12 Department of Pharmacy Practice, College of Pharmacy, Princess Nourah bint Abdulrahman University, Riyadh, Saudi Arabia

**Keywords:** COVID-19, Moderna vaccination, mortality, retrospective study, vaccination

## Abstract

**Background:**

COVID-19 remains associated with significant morbidity and mortality, particularly among hospitalized patients. Vaccination has been shown to reduce disease severity; however, real-world data comparing outcomes between vaccinated and unvaccinated patients and among different vaccine types remain limited.

**Methods:**

This study included 478 hospitalized patients with suspected or confirmed COVID-19. Demographic characteristics, vaccination status, comorbidities, disease severity, clinical outcomes, and mortality were assessed. Comparative analyses were performed between vaccinated and unvaccinated patients. Multivariable logistic regression was conducted to identify independent predictors of survival. A head-to-head comparison evaluated the impact of different vaccine types on hospitalization and outcomes.

**Results:**

The mean age of patients was 60.63 ± 13.86 years, and 56.9% were female. Most patients were unvaccinated (74.9%). Overall mortality was 19.46%. Vaccinated patients demonstrated a significantly higher recovery rate (67.5% vs. 35.75%) and lower mortality (12.5% vs. 21.79%, *p* < 0.05) compared with unvaccinated patients. Disease severity was significantly lower among vaccinated patients, with a greater proportion requiring only nasal cannula or simple mask oxygen therapy (*p* < 0.001). Multivariable logistic regression identified milder disease severity, absence of comorbidities, and Moderna vaccination as independent predictors of survival, while the need for CPAP or mechanical ventilation was strongly associated with reduced survival. Head-to-head comparison among different vaccine types showed no significant differences in hospital stay duration, outcomes, mortality rates, PCR results, or disease severity.

**Conclusion:**

COVID-19 vaccination was associated with improved clinical outcomes, reduced disease severity, and lower mortality among hospitalized patients. Disease severity and comorbidity burden were the strongest predictors of survival. No significant differences were observed among vaccine types in clinical outcomes. These findings support the protective role of COVID-19 vaccination in reducing severe disease and mortality.

## 1 Introduction

The coronavirus disease 2019 (COVID-19) pandemic has profoundly affected global health systems since its emergence in China in late 2019. Millions of healthcare workers have been directly impacted, and the disease has imposed an unprecedented burden on healthcare infrastructure worldwide (Hamdy et al., 2026). Although COVID-19 is primarily characterized by acute respiratory manifestations, accumulating evidence indicates that SARS-CoV-2 infection can adversely affect multiple organ systems, including the cardiovascular, renal, and gastrointestinal systems, contributing to increased morbidity and mortality among hospitalized patients (Zhou and Ye, 2026).

Given the rapid transmission and catastrophic consequences of the pandemic, the development and deployment of effective vaccines became a cornerstone in controlling the spread of COVID-19 (Dascalu et al., 2025). Governments, academic institutions, and the pharmaceutical industry mobilized at an unprecedented pace, resulting in the emergency authorization and global distribution of COVID-19 vaccines within less than 1 year of the pandemic declaration—an extraordinary milestone in modern medical history (Daems and Maes, 2022). These vaccination campaigns aimed not only to reduce infection rates but also to mitigate disease severity, decrease hospitalization, and lower mortality (Nachlis and Thomson, 2024).

Despite the proven benefits of vaccination, vaccine acceptance has remained a significant challenge. In 2019, the World Health Organization identified vaccine hesitancy as one of the top global threats to public health, defining it as the delay in acceptance or refusal of vaccines despite their availability (Peters, 2022). Concerns regarding vaccine safety, accelerated development timelines, and potential adverse effects have contributed to hesitancy, particularly among vulnerable populations (Dubé et al., 2021). Consequently, numerous clinical and observational studies have been conducted to assess the safety profiles and real-world effectiveness of COVID-19 vaccines (Liu et al., 2021; Zheng et al., 2022).

The literature presents conflicting findings regarding vaccine-related adverse outcomes. Some population-based studies, including reports from Taiwan, suggested an increased number of deaths temporally associated with vaccination, particularly among elderly individuals with multiple chronic or cardiovascular comorbidities, especially during early phases of vaccine rollout in high-risk populations such as hemodialysis patients and residents of long-term care facilities (Liu et al., 2021; Lu et al., 2024). These findings raised public concern; however, causal relationships remain debated.

Conversely, other studies have demonstrated favorable safety and tolerability profiles across different vaccine platforms. A study conducted among healthcare professionals in Tehran, who received two doses of Sputnik, Covaxin, AstraZeneca, or Sinopharm vaccines, reported that adverse effects were generally mild and self-limiting. Age was not significantly associated with side-effect incidence for AstraZeneca or Sputnik vaccines, although younger individuals reported higher rates of side effects with Sinopharm (Abdollahi et al., 2023). Overall, these findings support the safety of the available vaccines.

Traditionally, vaccine development requires 10–15 years to progress from discovery to large-scale production. The accelerated development of COVID-19 vaccines, while essential, intensified public scrutiny regarding their efficacy and long-term safety (Kashte et al., 2021). Currently approved COVID-19 vaccines utilize diverse platforms, including inactivated virus, viral vector, protein subunit, and nucleic acid–based technologies. Understanding whether these different vaccine types exert varying impacts on disease severity and outcomes remains an important clinical and public health question (Kudlay and Svistunov, 2022).

Several large-scale studies from the United States and the United Kingdom have demonstrated that COVID-19 vaccination significantly reduces hospitalization rates, disease severity, and mortality (Bernal et al., 2021; Whitaker et al., 2023). However, the pandemic trajectory in Egypt has differed from that reported in many Western countries, partly due to disparities between actual disease burden and officially reported cases and deaths (Medhat and El Kassas, 2020). Moreover, real-world data assessing the impact of vaccination on hospitalized patients in Egypt—particularly comparisons between vaccinated and unvaccinated individuals and between different vaccine types—remain limited.

Hospitalized COVID-19 patients represent a critical population for evaluating vaccine effectiveness, as disease severity, need for respiratory support, length of hospital stay, and survival outcomes can be directly assessed (Mohammed et al., 2022). Identifying independent predictors of survival, including vaccination status, vaccine type, comorbidity burden, and severity indicators such as oxygen and ventilatory requirements, is essential for optimizing patient management and guiding vaccination strategies (Tenforde et al., 2021).

Therefore, the aim of the present retrospective clinical study was to assess the impact of prior COVID-19 vaccination on morbidity, length of hospital stays, disease severity, and mortality among hospitalized patients in general hospitals in Egypt, to compare outcomes between vaccinated and unvaccinated individuals, to evaluate head-to-head differences among vaccine types, and to identify independent predictors of survival using multivariable logistic regression analysis.

## 2 Patients and methods

### 2.1 Study design and setting

This study was designed as a retrospective cohort study involving hospitalized patients with clinically and/or laboratory confirmed COVID-19 infection. The study was conducted in four hospitals located in the Giza Governorate, Egypt, including three Central Hospitals and one General Hospital. Data were collected over a six-month period, from September 2021 to March 2022, corresponding to a peak phase of COVID-19 hospital admissions in Egypt.

The study protocol was reviewed and approved by the Research Ethics Committee of the Central Directorate of Research and Health Development, with approval number 19–2022/21. All study procedures were conducted in accordance with the ethical principles outlined in the Declaration of Helsinki.

### 2.2 Study participants

**Figure 1:**
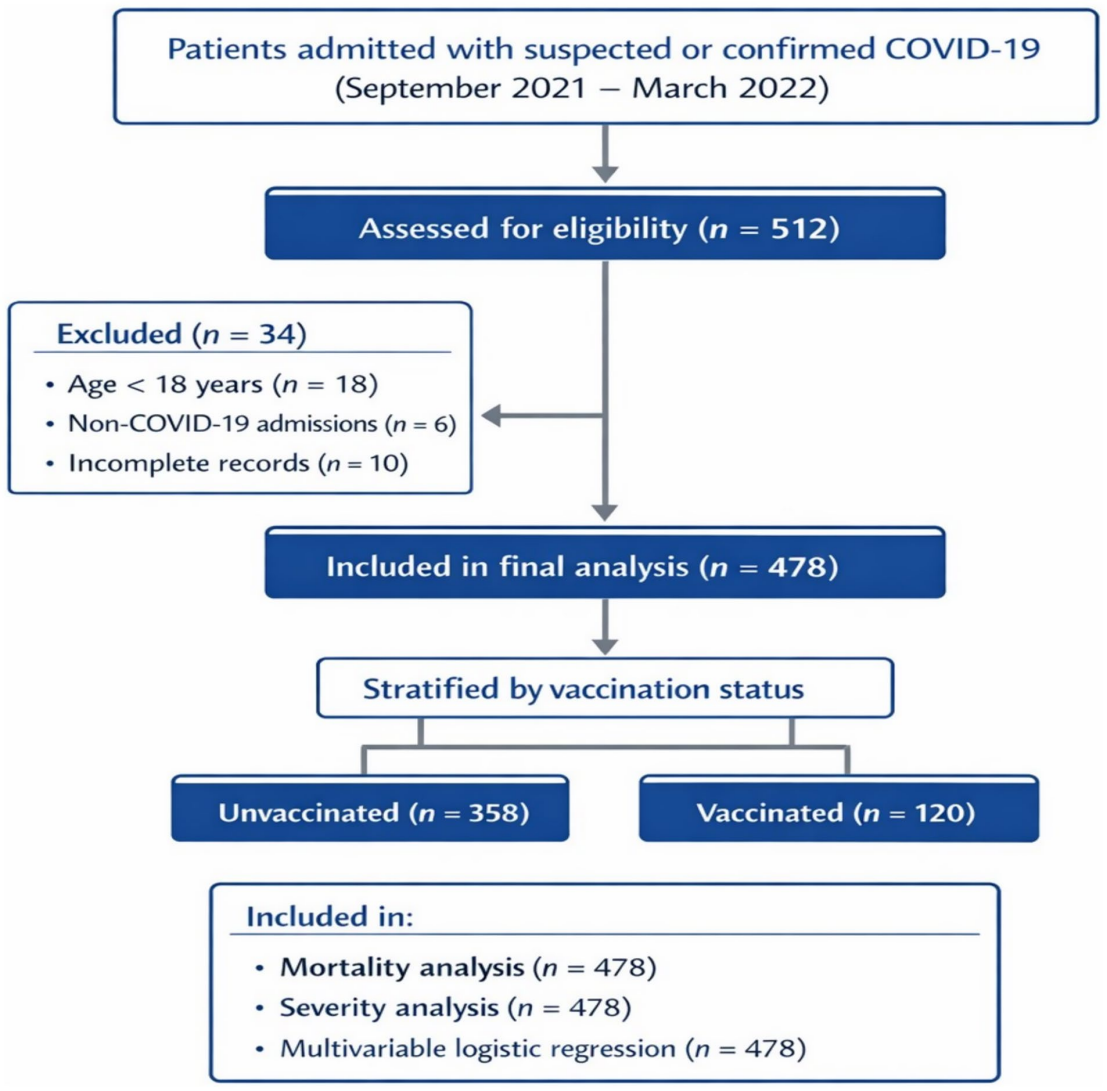
Flow diagram of patient inclusion and exclusion in the retrospective COVID-19 cohort study.

Eligible participants were adults aged ≥18 years who were admitted with clinically and/or laboratory-confirmed COVID-19 infection between September 2021 and March 2022 as shown in Figure 1. Laboratory confirmation was defined as a positive reverse transcription–polymerase chain reaction (RT-PCR) test for SARS-CoV-2. In cases where RT-PCR results were negative or unavailable, patients were included if they presented with clinical features highly suggestive of COVID-19 infection and had characteristic radiological findings on chest imaging (chest X-ray and/or computed tomography), in accordance with the Egyptian Ministry of Health national diagnostic protocol during the study period. Such patients were managed as confirmed COVID-19 cases during hospitalization.

Exclusion criteria included:

- Patients younger than 18 years,
- Patients admitted for non-COVID-19–related conditions,
- Patients with incomplete medical records regarding vaccination status or survival outcome.

All patients admitted to medical wards and ICUs with a diagnosis of COVID-19 during the study period were considered for inclusion.

### 2.3 Vaccination status assessment

Vaccination status was verified through collaboration with the Vaccination Registry Centers in each participating hospital. Patient national identification numbers were used to confirm prior COVID-19 vaccination status, including whether patients were vaccinated or unvaccinated, as well as the type of vaccine received. Documented vaccines included Pfizer-BioNTech, Sinophac, AstraZeneca, Sinopharm, Johnson & Johnson, Moderna, and Sputnik, in addition to mixed vaccine regimens. Information on dates of primary and booster doses was also collected when available.

### 2.4 Ethical considerations and informed consent

The study protocol was reviewed and approved by the Research Ethics Committee of the Central Directorate of Research and Health Development (Approval No. 19–2022/21). The research was conducted in accordance with the ethical standards of the institutional and national research committees and with the 2013 revision of the Declaration of Helsinki. As this was a retrospective study, formal written informed consent was waived; however, all data were anonymized and handled with strict confidentiality to protect patient privacy.

### 2.5 Data collection

Data were retrospectively extracted from patients’ medical records using a standardized data collection form. Collected variables included:

- Demographic data: age and gender
- Clinical data: presence of comorbidities (e.g., diabetes mellitus, hypertension, cardiovascular disease, chronic kidney disease, respiratory diseases)
- Administrative and hospitalization data: date of admission, date of discharge, length of hospital stay, ward or ICU admission
- COVID-19-related data: RT-PCR results, disease severity during hospitalization, oxygen and ventilatory support requirements
- Vaccination data: vaccination status (vaccinated or unvaccinated), vaccine type, and vaccination schedule when available
- Clinical outcomes: recovery, mortality, discharge against medical advice, transfer to another hospital, need for ICU admission, and disease severity indicators during hospital stay

### 2.6 Outcome measures

The primary outcome was defined as in-hospital mortality, referring to death occurring during the index hospitalization. Patients who were discharged against medical advice (DAMA) or transferred to another hospital were classified as alive at the time of discharge from the index hospital, provided that no death was documented prior to discharge. Due to the retrospective design and absence of centralized follow-up records, post-discharge outcomes for these patients were not available. Therefore, survival analysis was restricted to in-hospital mortality.

Secondary outcomes included disease severity during hospitalization, which was classified according to the highest level of respiratory support required during the hospital stay. Patients were categorized as having mild to moderate disease if they required only nasal cannula or simple face mask oxygen therapy, severe disease if they required oxygen via mask with reservoir, and critical disease if they required continuous positive airway pressure (CPAP) or invasive mechanical ventilation (MV). Length of hospital stay was calculated as the total number of days from hospital admission to discharge or death. Clinical outcome at discharge was categorized as recovery (defined as clinical improvement permitting discharge), death, discharge against medical advice (DAMA), or transfer to another hospital. Additionally, the need for ICU admission and advanced respiratory support (CPAP or MV) during hospitalization was recorded and analyzed.

### 2.7 Sample size calculation

All eligible patients admitted during the study period were included in the analysis. Post-hoc power calculation indicated that the sample size of 478 patients provided sufficient power (>80%) to detect a moderate difference in mortality (Cohen’s h = 0.35) between vaccinated and unvaccinated groups.

### 2.8 Statistical analysis

Data were organized, coded, and statistically analyzed using SPSS software version 27 (Statistical Package for the Social Sciences; SPSS Inc., Chicago, IL, United States). Quantitative variables were expressed as range, mean, and standard deviation (SD), while qualitative variables were presented as frequencies and percentages. Normality of numerical variables was assessed using the Kolmogorov–Smirnov test. As variables were normally distributed, comparisons between two independent groups were performed using the Student’s t-test. Categorical variables were compared using the Chi-square (χ^2^) test. Fisher’s exact test was used for categorical comparisons when expected cell counts were <5. A multivariable logistic regression analysis was conducted to identify independent predictors of survival, with adjustment for age and gender. Results were expressed as odds ratios (ORs) with 95% confidence intervals (CIs). All statistical tests were two-sided, and a *p*-value < 0.05 was considered statistically significant.

### 2.9 Missing data handling

Missing data were assessed for all key study variables prior to statistical analysis. The primary outcome (in-hospital mortality), vaccination status, disease severity, comorbidity status, age, gender, and length of hospital stay were complete for all included patients (*n* = 478). PCR testing results were unavailable for 160 patients (33.47%). No imputation methods were applied. Analyses were conducted using complete-case data for each variable. Because the primary outcome and exposure variables were complete, no patients were excluded from regression analysis due to missing data.

## 3 Results

### 3.1 Baseline characteristics and clinical profile of the studied patients

A total of 478 patients were included in the baseline assessment as shown in Table 1. The patients’ ages ranged from 22 to 98 years, with a mean age of 60.63 ± 13.86 years. More than half of the patients were younger than 65 years (278 patients, 58.16%), while 41.84% (200 patients) were aged 65 years or older.

**Table 1:** Variables assessments among the studied patients at baseline (*N* = 478).

Variables among groups				
Age (Years)	Range	22	-	98
	Mean ±SD	60.626	±	13.855
		N		%
Age group	< 65 Years	278		58.16
	≥65 Years	200		41.84
Gender	Male	206		43.10
	Female	272		56.90
If female patient	Non-pregnant	267		98.16
	Pregnant	5		1.84
Vaccinated or not??	Not vaccinated	358		74.90
	Vaccinated	120		25.10
Type of vaccination	Pfizer	16		13.33
	Astrazenica	27		22.50
	Sinophac	30		25.00
	Sinopharm	16		13.33
	Johnson	3		2.50
	Sputnik	3		2.50
	Moderna	11		9.17
	Mixed types	14		11.67
Length of hospital stay (Days)	Range	1	-	34
	Mean ±SD	5.854	±	4.919
Length of hospital stay	< 7 Days	307		64.23
	7–15 Days	150		31.38
	> 15 Days	21		4.39
Outcome	Death	93		19.46
	Recovery	209		43.72
	DAMA	73		15.27
	Transferred to another hospital	103		21.55
Mortality	Alive	385		80.54
	Death	93		19.46
Prevalence rate of Covid-19 infection (PCR results)	Not known	160		33.47
	Positive to covid 19	254		53.14
	Negative to covid 19	64		13.39
Comorbidity	Diabetes	213		44.56
	Hypertension	199		41.63
	Chronic Asthma	16		3.35
	CKD	17		3.56
	Hepatic	13		2.72
	Cardiovascular diseases	78		16.32
	COPD	12		2.51
	Previous stroke	12		2.51
	Other respiratory diseases	28		5.86
	Immunosuppressive	1		0.21
	Others comorbidity	61		12.76
Morbidity (Severity of disease along hospital stay)	Patient only needed nasal or simple mask	263		55.02
	Patient needed mask reservoir	144		30.13
	Patient needed MV	50		10.46
	Patient needed CPAP	21		4.39

Data presented as mean ± SD. DAMA, discharge against medical advice; CKD, chronic kidney diseases; COPD, chronic obstructive pulmonary diseases; MV, mechanical ventilation; CPAP, continuous positive airway pressure, *Significant (*p* < 0.05).

Regarding gender distribution, 56.90% of the patients were female (*n* = 272), and 43.10% were male (*n* = 206). Among female patients, the vast majority were non-pregnant (267 patients, 98.16%), whereas only 1.84% (*n* = 5) were pregnant.

Concerning COVID-19 vaccination status, 74.90% of the patients (*n* = 358) were not vaccinated, while 25.10% (*n* = 120) had received vaccination. Among vaccinated patients, Sinophac was the most frequently reported vaccine (25.00%), followed by AstraZeneca (22.50%), Pfizer and Sinopharm (each 13.33%), Mixed vaccine types (11.67%), Moderna (9.17%), and Johnson and Sputnik vaccines (each 2.50%).

The length of hospital stay ranged from 1 to 34 days, with a mean duration of 5.85 ± 4.92 days. Most patients had a hospital stay of less than 7 days (307 patients, 64.23%), followed by 7–15 days in 31.38% (*n* = 150), while only 4.39% (*n* = 21) stayed for more than 15 days.

In terms of patient outcomes, 43.72% of patients (*n* = 209) recovered, 19.46% (*n* = 93) died, 15.27% (*n* = 73) were discharged against medical advice (DAMA), and 21.55% (*n* = 103) were transferred to another hospital. Overall, 80.54% of patients were alive, whereas the mortality rate was 19.46%.

PCR testing for COVID-19 revealed that 53.14% of patients (*n* = 254) were PCR-positive, 13.39% (*n* = 64) were PCR-negative, while PCR results were not known for 33.47% of patients (*n* = 160).

Regarding comorbidities, diabetes mellitus was the most prevalent condition (44.56%), followed by hypertension (41.63%) and cardiovascular diseases (16.32%). Other reported comorbidities included other comorbid conditions (12.76%), other respiratory diseases (5.86%), chronic kidney disease (3.56%), chronic asthma (3.35%), hepatic diseases (2.72%), COPD (2.51%), previous stroke (2.51%), and immunosuppressive conditions (0.21%).

With respect to disease severity during hospital stay, more than half of the patients (55.02%, *n* = 263) required only nasal cannula or simple face mask oxygen therapy. Additionally, 30.13% (*n* = 144) required a mask with reservoir, 10.46% (*n* = 50) required mechanical ventilation, and 4.39% (*n* = 21) required continuous positive airway pressure (CPAP) support.

### 3.2 Comparison of clinical and demographic characteristics between vaccinated and unvaccinated patients

A comparative analysis was conducted between vaccinated (*n* = 120) and unvaccinated (*n* = 358) patients to evaluate differences in demographic characteristics, clinical outcomes, comorbidities, and disease severity as shown in Table 2.

**Table 2:** Variables assessments among the studied vaccinated and unvaccinated patients (*N* = 478).

Variables		Patients						T-test	
		Unvaccinated			Vaccinated			t	*p*-value
Age (Years)	Range	22	-	97	24	-	98	0.031	0.975
	Mean ±SD	60.637	±	14.128	60.592	±	13.061		
Chi-square		N		%	N		%	X^2^	*p*-value
Age group	< 65 Years	204		56.98	74		61.67	0.810	0.368
	> = 65 Years	154		43.02	46		38.33		
Gender	Male	152		42.46	54		45.00	0.237	0.627
	Female	206		57.54	66		55.00		
If female patient	Non-pregnant	201		97.57	66		100.00	1.632	0.201
	Pregnant	5		2.43	0		0.00		
T-test								t	*p*-value
Length of hospital stay (Days)	Range	1	-	34	1	-	22	−0.634	0.527
	Mean ±SD	5.771	±	5.146	6.100	±	4.180		
Chi-square		N		%	N		%	X^2^	*p*-value
Length of hospital stay	< 7 Days	235		65.64	72		60.00	1.476	0.478
	7–15 Days	107		29.89	43		35.83		
	> 15 Days	16		4.47	5		4.17		
Outcomes	Death	78		21.79	15		12.50	37.532	<0.001*
	Recovery	128		35.75	81		67.50		
	DAMA	61		17.04	12		10.00		
	Transferred to another hospital	91		25.42	12		10.00		
Mortality	Alive	280		78.21	105		87.50	4.947	0.026*
	Death	78		21.79	15		12.50		
Prevalence rate of Covid-19 infection (PCR results)	Not known	131		36.59	29		24.17	10.881	0.004*
	Positive to covid 19	188		52.51	66		55.00		
	Negative to covid 19	39		10.89	25		20.83		
Comorbidity	Diabetes	149		41.62	64		53.33	4.991	0.025*
	Hypertension	139		38.83	60		50.00	4.617	0.032*
	Chronic Asthma	12		3.35	4		3.33	0.000	0.992
	CKD	13		3.63	4		3.33	0.023	0.879
	Hepatic	9		2.51	4		3.33	0.228	0.633
	Cardiovascular diseases	54		15.08	24		20.00	1.591	0.207
	COPD	8		2.23	4		3.33	0.443	0.506
	Previous stroke	10		2.79	2		1.67	0.466	0.495
	Other respiratory diseases	19		5.31	9		7.50	0.784	0.376
	Immunosuppressive	1		0.28	0		0.00	0.336	0.562
	Others comorbidity	50		13.97	11		9.17	1.860	0.173
(Morbidity) Severity of disease along hospital stay	Patient only needed nasal or simple mask	178		49.72	85		70.83	18.935	<0.001*
	Patient needed mask reservoir	120		33.52	24		20.00		
	Patient needed MV	45		12.57	5		4.17		
	Patient needed CPAP	15		4.19	6		5.00		

Data presented as mean ± SD, numbers, and percentages. DAMA, discharge against medical advice; CKD, chronic kidney diseases; COPD, chronic obstructive pulmonary diseases; MV, mechanical ventilation; CPAP, continuous positive airway pressure; X^2^, Chi-square; *Significant (*p* < 0.05).

There was no significant difference in age between the two groups. The mean age of unvaccinated patients was 60.64 ± 14.13 years, compared with 60.59 ± 13.06 years in vaccinated patients (*p* = 0.975). Similarly, age group distribution did not differ significantly, with patients aged <65 years representing 56.98% of the unvaccinated group and 61.67% of the vaccinated group (*p* = 0.368).

Gender distribution was also comparable between groups, as males constituted 42.46% of unvaccinated patients and 45.00% of vaccinated patients (*p* = 0.627). Among female patients, pregnancy status showed no statistically significant difference, with 97.57% of unvaccinated females being non-pregnant compared to 100% in the vaccinated group (*p* = 0.201).

Regarding hospitalization duration, there was no significant difference in the mean length of hospital stay, which was 5.77 ± 5.15 days in unvaccinated patients and 6.10 ± 4.18 days in vaccinated patients (*p* = 0.527). Likewise, categorical analysis of hospital stay duration showed no significant difference between the two groups (*p* = 0.478).

In contrast, clinical outcomes differed significantly between vaccinated and unvaccinated patients (*p* < 0.001). The recovery rate was markedly higher among vaccinated patients (67.50%) compared to unvaccinated patients (35.75%). Conversely, mortality was significantly lower in vaccinated patients (12.50%) than in unvaccinated patients (21.79%). Additionally, unvaccinated patients had higher rates of discharge against medical advice (17.04%) and transfer to another hospital (25.42%) compared with vaccinated patients (10.00% for each).

Overall mortality analysis confirmed a significantly higher survival rate among vaccinated patients (87.50%) compared to unvaccinated patients (78.21%, *p* = 0.026).

PCR testing results for COVID-19 infection differed significantly between groups (*p* = 0.004). A higher proportion of vaccinated patients tested PCR-negative (20.83%) compared with unvaccinated patients (10.89%), while PCR results were unknown more frequently among unvaccinated patients (36.59% vs. 24.17%).

With respect to comorbidities, diabetes mellitus and hypertension were significantly more prevalent among vaccinated patients (53.33 and 50.00%, respectively) compared to unvaccinated patients (41.62 and 38.83%; *p* = 0.025 and *p* = 0.032, respectively). No statistically significant differences were observed between groups for other comorbid conditions.

Analysis of disease severity during hospitalization revealed significant differences between groups (*p* < 0.001). A substantially higher proportion of vaccinated patients (70.83%) required only nasal cannula or simple face mask oxygen therapy, compared to 49.72% of unvaccinated patients. In contrast, the need for mechanical ventilation was markedly higher among unvaccinated patients (12.57%) than vaccinated patients (4.17%), indicating a more severe disease course in the unvaccinated group.

### 3.3 Multivariable logistic regression analysis of factors affecting survival

A multivariable logistic regression analysis was performed to identify independent factors associated with patient survival among the studied population (*N* = 478) as shown in Table 3.

**Table 3:** Multivariable logistic regression of factors affecting survival rate (*N* = 478).

Variables	Odds ratio (OR)	95% C. I.	
		Lower	Upper
Age	1.013	0.987	1.040
Male Gender	0.937	0.468	1.878
Nasal or Simple Mask*	4.514	2.096	9.721
CPAP or MV*	0.003	0.000	0.027
No Comorbidities*	2.747	1.110	6.802
Moderna (mRNA-1273) vaccination*	0.120	0.039	0.371
Vaccinated or not	1.063	0.468	2.414

Data presented by OR, odds ratio with 95%; CI, confidence interval; MV, mechanical ventilation; CPAP, continuous positive airway pressure.

Increasing age was not significantly associated with survival (OR = 1.013, 95% CI: 0.987–1.040). Similarly, male gender showed no significant effect on survival outcomes (OR = 0.937, 95% CI: 0.468–1.878).

Patients who required only nasal cannula or simple face mask oxygen therapy had a significantly higher likelihood of survival, with an odds ratio of 4.51 (95% CI: 2.10–9.72). In contrast, patients who required CPAP or mechanical ventilation had a markedly reduced survival probability (OR = 0.003, 95% CI: 0.000–0.027).

The absence of comorbidities was independently associated with improved survival, as patients without comorbid conditions had 2.75-fold higher odds of survival (OR = 2.747, 95% CI: 1.110–6.802).

Regarding vaccination, receipt of the Moderna vaccine (mRNA-1273) was significantly associated with survival, showing a strong protective effect (OR = 0.120, 95% CI: 0.039–0.371). However, vaccination status overall (vaccinated versus unvaccinated) was not independently associated with survival after adjustment for other variables (OR = 1.063, 95% CI: 0.468–2.414).

In the multivariable logistic model (age, sex, comorbidity count, severity, PCR, vaccination), severity was the strongest determinant of death (vs Mild: Moderate OR 4.317, 95% CI 1.988–9.375; Severe OR 20.856, 95% CI 7.104–61.229; Critical OR 1382.248, 95% CI 168.318–11351.219; all *p* < 0.001). Vaccination was not independently associated with mortality (OR 0.945, 95% CI 0.417–2.142; *p* = 0.892).

Propensity Score Matching (PSM) analysis. Propensity score matching on age, sex, comorbidity count, severity, PCR produced 93 matched pairs. Balance improved with residual imbalance on a few covariates (maximum |SMD| ≈ 0.217). In the matched cohort, mortality was 11.7% in both vaccinated and unvaccinated groups (risk difference 0.0; risk ratio 1.0).

Overall, disease severity indicators and absence of comorbidities emerged as the most influential independent predictors of survival in the multivariable model.

### 3.4 Head-to-head comparison of different COVID-19 vaccine types and clinical outcomes

A head-to-head comparison was performed to evaluate the association between different COVID-19 vaccine types and length of hospital stay, clinical outcomes, mortality, PCR results, and disease severity among the studied patients as shown in Table 4.

**Table 4:** Head-to-head comparison of the effect of different vaccines types on hospitalization, outcomes, mortality rate among the studied patients (*N* = 478).

Variables		Type of vaccination																Chi-square	
		Pfizer (BNT162b2)		AstraZeneca (ChAdOx1 nCoV-19)		Sinophac (CoronaVac)		Sinopharm (BBIBP-CorV)		Johnson (Ad26. COV2. S)		Sputnik (Gam-COVID-Vac)		Moderna (mRNA-1273)		Mixed types			
		N	%	N	%	N	%	N	%	N	%	N	%	N	%	N	%	X^2^	*p*-value
Length of hospital stay	< 7 Days	10	62.50	16	59.26	19	63.33	10	62.50	1	33.33	2	66.67	4	36.36	10	71.43	17.490	0.231
	7–15 Days	6	37.50	7	25.93	11	36.67	6	37.50	2	66.67	1	33.33	7	63.64	3	21.43		
	> 15 Days	0	0.00	4	14.81	0	0.00	0	0.00	0	0.00	0	0.00	0	0.00	1	7.14		
Outcomes	Death	2	12.50	4	14.81	2	6.67	3	18.75	0	0.00	1	33.33	1	9.09	2	14.29	12.998	0.909
	Recovery	10	62.50	17	62.96	23	76.67	9	56.25	2	66.67	2	66.67	7	63.64	11	78.57		
	DAMA	3	18.75	4	14.81	1	3.33	2	12.50	0	0.00	0	0.00	1	9.09	1	7.14		
	Transferred to another hospital	1	6.25	2	7.41	4	13.33	2	12.50	1	33.33	0	0.00	2	18.18	0	0.00		
Mortality rate	Alive	14	87.50	23	85.19	28	93.33	13	81.25	3	100.0	2	66.67	10	90.91	12	85.71	3.414	0.844
	Death	2	12.50	4	14.81	2	6.67	3	18.75	0	0.00	1	33.33	1	9.09	2	14.29		
Prevalence rate of Covid-19 infection (PCR results)	Not known	7	43.75	4	14.81	7	23.33	3	18.75	0	0.00	2	66.67	1	9.09	5	35.71	20.339	0.120
	Positive to covid 19	7	43.75	13	48.15	16	53.33	11	68.75	3	100.0	0	0.00	9	81.82	7	50.00		
	Negative to covid 19	2	12.50	10	37.04	7	23.33	2	12.50	0	0.00	1	33.33	1	9.09	2	14.29		
Morbidity (Severity of disease along hospital stay)	Patient only needed nasal or simple mask	13	81.25	18	66.67	22	73.33	10	62.50	2	66.67	1	33.33	7	63.64	12	85.71	17.570	0.676
	Patient needed mask reservoir	3	18.75	6	22.22	5	16.67	3	18.75	1	33.33	1	33.33	4	36.36	1	7.14		
	Patient needed MV	0	0.00	1	3.70	1	3.33	1	6.25	0	0.00	1	33.33	0	0.00	1	7.14		
	Patient needed CPAP	0	0.00	2	7.41	2	6.67	2	12.50	0	0.00	0	0.00	0	0.00	0	0.00		

Data presented as numbers and percentages, DAMA, discharge against medical advice; CKD, chronic kidney diseases; COPD, chronic obstructive pulmonary diseases; MV, mechanical ventilation; CPAP, continuous positive airway pressure; X^2^, Chi-square; *Significant (*p* < 0.05).

Regarding the length of hospital stay, no statistically significant differences were observed among the different vaccine types (*p* = 0.231). Across all vaccine groups, the majority of patients were hospitalized for less than 7 days, with proportions ranging from 33.33% in Johnson recipients to 71.43% among those who received mixed vaccine types.

Analysis of clinical outcomes demonstrated no significant differences between vaccine groups (*p* = 0.909). Recovery was the most common outcome across all vaccine types, ranging from 56.25% in Sinopharm (BBIBP-CorV) recipients to 78.57% in those receiving mixed vaccines. Mortality rates within outcome categories were generally low and varied across vaccine types without reaching statistical significance.

Similarly, comparison of the overall mortality rate showed no significant variation among vaccine types (*p* = 0.844). Survival rates were high across all groups, ranging from 66.67% in Sputnik (Gam-COVID-Vac) recipients to 100% in Johnson vaccine recipients.

The prevalence of COVID-19 infection based on PCR testing did not differ significantly among vaccine types (*p* = 0.120). PCR positivity was observed across all vaccine groups, with the highest proportion among Johnson (Ad26. COV2. S) vaccine recipients (100%), while PCR-negative results ranged between 9.09 and 37.04% across groups.

With respect to disease severity during hospital stay, there were no statistically significant differences among vaccine types (*p* = 0.676). Most patients in all vaccine groups required only nasal cannula or simple face mask oxygen therapy, with proportions ranging from 33.33% in Sputnik (Gam-COVID-Vac) recipients to 85.71% among those who received mixed vaccine types. The need for mechanical ventilation or CPAP was infrequent and did not significantly differ between vaccine categories.

Overall, the head-to-head comparison demonstrated that no individual COVID-19 vaccine type was associated with significant differences in hospitalization duration, clinical outcomes, mortality, PCR results, or disease severity among the studied patients.

Data completeness was high for all primary and secondary outcome variables. There were no missing data for in-hospital mortality, vaccination status, age, gender, comorbidities, disease severity, or length of hospital stay. PCR results were unavailable in 160 patients (33.47%), reflecting real-world diagnostic limitations during peak pandemic waves.

## 4 Discussion

The present study provides robust real-world evidence regarding the impact of COVID-19 vaccination on hospitalized patients in Egypt, analyzing morbidity, mortality, disease severity, and the influence of different vaccine types. Among 478 patients admitted to general hospitals, only 24.7% had received any COVID-19 vaccination prior to hospitalization, reflecting a substantial gap in immunization among high-risk populations. This low vaccination uptake is concerning, particularly in light of evidence that vaccination significantly reduces the risk of severe disease and death (Huang and Kuan, 2022). Similar trends have been reported in other studies of critically ill patients, where low vaccination coverage has been associated with increased ICU admissions and mortality (Havaldar and Selvam, 2024; Maleki et al., 2025), highlighting the ongoing need for targeted public health interventions to improve vaccine accessibility and acceptance. In a propensity-matched observational analysis, vaccinated individuals with breakthrough infections had significantly lower rates of ICU admission and in-hospital mortality compared with matched unvaccinated controls, further supporting the role of vaccination in reducing critical-care burden (Maleki et al., 2025).

Vaccinated patients in our cohort demonstrated markedly improved outcomes. Mortality among vaccinated patients was 12.5%, compared to 21.8% among unvaccinated patients, while recovery rates were significantly higher in the vaccinated group. Moreover, vaccinated individuals required less intensive respiratory support, with over 70% requiring only nasal or simple mask oxygen therapy, compared to 49.7% of unvaccinated patients. These findings corroborate prior global evidence, confirming that COVID-19 vaccines, regardless of platform, provide substantial protection against severe disease and death. A multicenter cohort study of critically ill COVID-19 patients found that vaccinated individuals were significantly less likely to experience ICU mortality compared with unvaccinated patients, indicating that vaccination reduces the risk of death even among severe cases (Havaldar and Selvam, 2024). A large observational study reported that full vaccination was associated with a significantly lower risk of ICU admission, lower mortality risk, and shorter hospital stays among hospitalized COVID-19 patients, clearly illustrating the benefit of vaccination in preventing progression to critical illness (Maleki et al., 2025).

Mechanistically, vaccination reduces disease severity and mortality through a combination of humoral and cellular immune responses (Speiser and Bachmann, 2020). Humoral immunity primarily involves the production of neutralizing antibodies that specifically target the spike protein of SARS-CoV-2, preventing viral entry into host cells and limiting initial viral replication (Carrillo et al., 2021). The quantity and quality of these antibodies are influenced by the type of vaccine administered, the number of doses, and the time elapsed since vaccination (Shah et al., 2020). mRNA vaccines such as Moderna and Pfizer have been shown to elicit higher neutralizing antibody titers compared to inactivated or vector-based vaccines (Tada et al., 2021), which may partly explain the lower rates of severe disease and mortality observed among patients who received these vaccines in our cohort.

Beyond neutralizing antibodies, vaccines stimulate cell-mediated immunity, which is crucial for controlling viral infections once cells are infected. CD8 + cytotoxic T lymphocytes recognize and destroy infected host cells, limiting viral replication and tissue damage, while CD4 + helper T cells coordinate the immune response by supporting antibody production and enhancing cytotoxic responses (Zhuang et al., 2024). This dual mechanism not only prevents the progression of mild infections to severe disease but also mitigates hyperinflammatory responses, such as cytokine storms, that are strongly associated with respiratory failure and multi-organ dysfunction in severe COVID-19 (Janssens et al., 2022).

Vaccination also promotes the formation of memory B and T cells, which provide long-term immune surveillance and allow for a rapid and robust response upon subsequent exposure to SARS-CoV-2 or its variants (Sette and Crotty, 2022). This immunological memory is particularly important in preventing severe disease even when breakthrough infections occur, as it can rapidly contain viral replication and reduce tissue damage. However, the strength and durability of these immune responses may vary among individuals, depending on factors such as age, comorbidities, and immunosuppressive conditions (Hartley et al., 2022). Elderly patients and those with chronic illnesses may have reduced vaccine-induced immunity, explaining why some vaccinated individuals in our study still required supplemental oxygen or advanced respiratory support (Villar-Álvarez et al., 2022).

In addition, SARS-CoV-2 variants with mutations in the spike protein can partially evade vaccine-induced neutralizing antibodies, potentially leading to breakthrough infections. Despite this, vaccines still confer substantial protection against severe outcomes, likely due to the broader cellular immune response that recognizes conserved viral epitopes (Harvey et al., 2021). This explains why, even in the presence of variants, vaccinated patients in our cohort experienced milder disease and lower mortality compared to unvaccinated individuals. Overall, these immunological mechanisms—neutralizing antibodies, T-cell-mediated cytotoxicity, and immunological memory—collectively reduce viral load, prevent extensive lung injury, and improve clinical outcomes.

The study also highlights the potential impact of vaccine type on clinical outcomes. Among the vaccinated cohort, mRNA vaccines, particularly Moderna (mRNA-1273), were associated with the lowest mortality and the least severe disease. Multivariable logistic regression demonstrated that Moderna (mRNA-1273) vaccination independently predicted improved survival (OR = 0.12, 95% CI: 0.039–0.371). While the small sample sizes for individual vaccine subgroups limit definitive conclusions, these results are consistent with studies indicating higher immunogenicity and neutralizing antibody titers elicited by mRNA vaccines compared to inactivated or vector-based vaccines (Sarker et al., 2022; Ben Ahmed et al., 2022). Inactivated virus vaccines, while effective at reducing hospitalization, may confer relatively lower neutralizing antibody titers and shorter duration of immunity (Law et al., 2023), which could explain the observed differences in outcomes among vaccine subtypes in our cohort.

Comparisons between vaccine types are limited by very small subgroup sizes and incomplete vaccination data (no dose number or timing), producing unstable and non-informative estimates. As such, vaccine-type differences should be viewed as descriptive observations only, not as evidence of true comparative effectiveness.

Comorbidities were prevalent in this cohort, with diabetes and hypertension being the most common. The average number of comorbidities was slightly higher among surviving patients (2.1) compared to deceased patients (2.0), indicating that underlying illnesses remain critical predictors of COVID-19 outcomes. Patients with multiple comorbidities have impaired immune responses and higher baseline inflammation, which may attenuate vaccine effectiveness. This emphasizes that while vaccination provides strong protection, comorbidity management, early clinical intervention, and booster doses remain essential to improving patient outcomes (Bonanni et al., 2023).

Another key finding is the continued requirement for supplemental oxygen in a subset of vaccinated patients. While vaccination clearly reduces mortality and disease severity, it does not completely eliminate the risk of severe infection, particularly in older patients or those with comorbidities. This aligns with global observations of breakthrough infections, where vaccinated individuals may still require hospitalization and oxygen support but generally experience milder disease, lower supplemental oxygen requirements, and reduced mortality compared with unvaccinated patients (Myers et al., 2022; Finn et al., 2024).

The study’s findings have important clinical and public health implications. Vaccination, particularly with mRNA vaccines, not only protects individual patients but also reduces the burden on hospital resources by lowering the proportion of patients requiring intensive respiratory support. From a policy perspective, these results support prioritizing mRNA vaccines for high-risk populations and underscore the importance of continuous public health messaging to improve vaccine uptake. Additionally, our data suggest that vaccination campaigns should target not only unvaccinated individuals but also those with comorbidities who may benefit from booster doses or enhanced monitoring.

This study had several strength points. This study is a real-world, multi-hospital cohort, which enhances the generalizability of our findings. Vaccination status was verified via the national registry using unique ID numbers, strengthening exposure classification and reducing the risk of misclassification. Furthermore, we report clinically relevant outcomes, including mortality, need for respiratory support, disease severity, and length of hospital stay, allowing meaningful interpretation of the results.

This retrospective study has several limitations. First, despite adjustment for age, sex, comorbidity burden, PCR status, and peak in-hospital severity, the risk of residual confounding remains, particularly regarding disease severity and healthcare-seeking behavior. Second, incomplete PCR confirmation in a subset of patients may have introduced case definition misclassification. Third, patients discharged against medical advice or transferred to other facilities were coded as alive due to unavailable post-discharge outcomes, which may underestimate true mortality. Fourth, detailed vaccination data—including number of doses, booster status, vaccination dates, and vaccine brand—were incomplete or limited, and several vaccine groups had small and uneven sample sizes. Consequently, vaccination variables were excluded from adjusted analyses, head-to-head comparisons between vaccine types should be interpreted cautiously, and no causal inference regarding vaccination or time since vaccination can be made. Fifth, missing data primarily affected PCR results; we applied complete case analysis, as missingness was limited and non-systematic, but some residual bias may remain. Finally, the retrospective design inherently carries the risk of selection and information bias, and the lack of post-discharge follow-up prevented assessment of long-term outcomes, readmissions, or post-discharge complications. Future prospective studies with larger, multicenter cohorts are warranted to validate these findings, clarify the impact of different vaccine types, and examine long-term outcomes post-vaccination.

In summary, this study demonstrates that COVID-19 vaccination significantly reduces mortality, disease severity, and the need for advanced respiratory support among hospitalized patients. Moderna vaccination, in particular, was associated with the most favorable outcomes, suggesting that vaccine platform may influence patient prognosis. Comorbidities remain important determinants of disease severity, highlighting the need for targeted vaccination strategies and continued monitoring of high-risk individuals. These findings reinforce the critical role of COVID-19 vaccination in mitigating morbidity and mortality and provide a framework for optimizing vaccine deployment to reduce the burden on healthcare systems.

## 5 Conclusion

In conclusion, this study demonstrates that COVID-19 vaccination significantly improves clinical outcomes among hospitalized patients, reducing mortality, disease severity, and the need for advanced respiratory support. The type of vaccine administered plays a crucial role, with mRNA vaccines—particularly Moderna—associated with the most favorable survival outcomes. Despite these benefits, breakthrough infections and the need for supplemental oxygen in some vaccinated patients highlight that vaccination mitigates but does not completely eliminate the risk of severe disease, especially in older adults and patients with comorbidities.

These findings underscore the critical importance of continued vaccination efforts, prioritization of high-risk populations, and tailored public health strategies to enhance vaccine uptake and protection. Additionally, the study highlights the need for ongoing monitoring of vaccine effectiveness, particularly in the context of emerging variants, and the implementation of booster doses to sustain immune protection. Ultimately, widespread vaccination remains a cornerstone in reducing the burden of COVID-19 on healthcare systems, improving patient prognosis, and controlling the pandemic.

## Data Availability

The raw data supporting the conclusions of this article will be made available by the authors, without undue reservation.
